# SIRT3 and Metabolic Reprogramming Mediate the Antiproliferative Effects of Whey in Human Colon Cancer Cells

**DOI:** 10.3390/cancers13205196

**Published:** 2021-10-16

**Authors:** Nunzia D’Onofrio, Elisa Martino, Anna Balestrieri, Luigi Mele, Gianluca Neglia, Maria Luisa Balestrieri, Giuseppe Campanile

**Affiliations:** 1 Department of Precision Medicine, University of Campania Luigi Vanvitelli, Via L. De Crecchio 7, 80138 Naples, Italy; nunzia.donofrio@unicampania.it (N.D.); elisa.martino@unicampania.it (E.M.); 2 Department of Animal Health, Istituto Zooprofilattico Sperimentale del Mezzogiorno, 80055 Portici, Italy; anna.balestrieri@izsmportici.it; 3 Department of Experimental Medicine, University of Campania Luigi Vanvitelli, Via Luciano Armanni 5, 80138 Naples, Italy; luigi.mele@unicampania.it; 4 Department of Veterinary Medicine and Animal Production, University of Naples Federico II, Via F. Delpino 1, 80137 Naples, Italy; neglia@unina.it (G.N.); giuseppe.campanile@unina.it (G.C.)

**Keywords:** whey, colon cancer, SIRT3, mitochondria, metabolism

## Abstract

**Simple Summary:**

Colorectal cancer (CRC) is the second leading cause of cancer-related deaths and the most frequently diagnosed cancer type. CRC risk can be preventable by modifiable risk factors, including diet. This study provides evidence on the antiproliferative and pro-apoptotic effects of whey from Mediterranean water buffalo (*Bubalus bubalis*) milk in HT-29, HCT 116, LoVo, and SW480 cells. Results showed that whey induced metabolic dysfunctions and modulated the bioenergetic signature of CRC cells by targeting SIRT3 expression. These findings unveil the anti-neoplastic effects of whey and pave the way for the use of this by-product, rich in bioactive nutrients, in the setting of novel prevention strategies to reduce the risk of CRC.

**Abstract:**

Emerging strategies to improve healthy aging include dietary interventions as a tool to promote health benefits and reduce the incidence of aging-related comorbidities. The health benefits of milk are also linked to its richness in betaines and short-chain acylcarnitines, which act synergistically in conferring anticancer, anti-inflammatory, and antioxidant properties. Whey, despite being a dairy by-product, still has a considerable content of bioactive betaines and acylcarnitines. Here, we investigated the anticancer properties of whey from Mediterranean water buffalo (*Bubalus bubalis*) milk by testing its antiproliferative effects in colorectal cancer (CRC) cells HT-29, HCT 116, LoVo and SW480. Results indicated that treatment with whey for 72 h inhibited cell proliferation (*p* < 0.001), induced cell cycle arrest, and apoptosis via caspase-3 activation, and modulated cell metabolism by limiting glucose uptake and interfering with mitochondrial energy metabolism with the highest effects observed in HT-29 and HCT 116 cells. At molecular level, these effects were accompanied by upregulation of sirtuin 3 (SIRT3) (*p* < 0.01) and peroxisome proliferator-activated receptor (PPAR)-γ expression (*p* < 0.001), and downregulation of lactate dehydrogenase A (LDHA) (*p* < 0.01), sterol regulatory-element binding protein 1 (SREBP1) (*p* < 0.05), and PPAR-α (*p* < 0.01). Transient SIRT3 gene silencing blocked the effects of whey on the LDHA, PPAR-γ, and PPAR-α protein expressions (*p* < 0.01) suggesting that the whey capacity of perturbating the metabolic homeostasis in CRC cell lines is mediated by SIRT3.

## 1. Introduction

In the nutritional field, the search of nutrients and bioactive food components capable of modifying the epigenetic phenomena affecting the hallmarks of aging has emerged as an attractive tool in the setting of dietary interventions to promote health benefits and decrease the incidence of aging-related comorbidities. One component of epigenetic regulation involves histone deacetylases, which include the histone deacetylases (class I, II, and IV) and sirtuin deacetylases (class III). In mammals, the SIRT family includes seven members (SIRT1-7) of nicotine adenine dinucleotide (NAD+)-dependent enzymes involved in the regulation of cell metabolism, oxidative stress, cell survival, DNA repair, and the nutrient-sensing pathways potentially affecting health span [1,2].

The health potential of milk has recently been brought to attention due to its content in δ-valerobetaine and the peculiar metabolomic profile, with regard to the milk from the Italian Mediterranean buffalo (*Bubalus bubalis*) particularly rich in l-carnitine, short-chain acylcarnitines, and betaines [3,4,5]. The antioxidant, anti-inflammatory and anticancer properties of δ-valerobetaine, acting in synergism with other betaines, occur in endothelial cells, colorectal cancer (CRC) cells and human oral squamous cell carcinoma cells through the modulation of sirtuins [6,7,8,9]. In CRC cells, LoVo and HT-29, the activation of SIRT6 mediated the anticancer and apoptotic activity of milk [7]. CRC is the third most common cancer throughout the world in terms of incidence, but second in terms of mortality, with 1.9 million cases and 935,000 deaths in 2020 [10]. As a marker of socioeconomic development, CRC incidence rates trend to increase with Human Development Index (HDI) in transition countries, rising by 1% to 4% per year in high-income countries [10]. The majority of cancer diseases, including CRC, are characterized by mitochondrial dysfunction and increased oxidative stress with mitochondrial dysfunction affecting a wide range of metabolic pathways [11,12,13]. Mitochondrial function and integrity are controlled by the mitochondria guardian SIRT3 which acts on numerous substrates to activate fat oxidation, amino acid metabolism and electron transport [14,15,16]. In SW620 CRC cells, SIRT3 silencing led to a decreased mitochondrial biogenesis, which in turn affected cell viability [17]. Clinical studies on association between SIRT3 and tumorigenesis showed controversial results. In hepatocellular carcinoma, SIRT3 upregulation re-sensitizes to sorafenib treatment [18]. Moreover, SIRT3 activation by the Bcl-2 inhibitor ABT737 contributes to improve cisplatin resistance in ovarian cancer [19]. On the contrary, in cervical cancer cells, the deacetylation of Acetyl-CoA Carboxylase by SIRT3 promotes lipid metabolism reprogramming, thus triggering cancer migration and invasion, displaying an oncogenic role [20]. Similarly, in diffuse large B cell lymphomas, SIRT3 increases the metabolism of TCA cycle by enhancing GDH activity to promote lymphomagenesis [21]. In SW480 and SW620 CRC cells, the mitochondrial nitric oxide synthase (NOS)/SIRT3/SOD2 axis regulates the reactive oxygen species (ROS) production to prevent apoptosis [22]. Indeed, although SIRT3 was reported to shift cellular metabolism toward increased glycolysis in some types of cancer, it acts as tumor suppressor by modulating ROS and limiting the oxidative damage in cellular components in other types of cancer [23,24,25,26]. A recent study showed that SIRT3 activates the serine hydroxymethyltransferase 2 (SHMT2) to promote colorectal carcinogenesis [27].

In addition to milk, whey obtained from the production of white cheeses, such as ricotta, following the removal of the protein fraction contains considerable amounts of betaines, l-carnitine and acylcarnitines. In particular, whey from Italian Mediterranean buffalo shows mean values of 41 mg/L of l-carnitine and acetyl-carnitine, 27 mg/L of propionyl-carnitine, 22 mg/L of δ-valerobetaine, and 10 mg/L of glycine betaine [28]. While whey protein supplementation is known to ameliorate the energy balance and the muscle mass in cancer patients [29,30,31,32,33], to date there is no evidence on the potential anticancer activity of the whey from Italian Mediterranean water buffalo.

In CRC cells, the chemopreventive effects of 3 kDa-fraction of milk and its biomolecules occur through mitochondrial dysfunction and downregulation of SIRT3 [9]. SIRT3-silenced metastatic CRC cells showed mitochondrial aggregation and ROS accumulation and colony formation ability leading to decreased mitochondrial dysfunction [9,17]. The imbalanced mitochondrial functions affected cell viability and expression of several proteins related to mitochondrial biogenesis, including the lactate dehydrogenase (LDH) expression [17]. Peroxisome proliferator activated receptors (PPAR), a nuclear ligand-activated transcription factors, act on CRC tumorigenesis [34]. Enhanced PPARγ partially contributed to the suppressed cell viability and induced apoptosis in SW948 colon cancer cells [35]. Although both SIRT3 and PPAR are considered attractive targets for therapies against CRC, their full detailed correlation is still not clear. Within this framework, the present study aimed at investigating the anticancer properties of 3 kDa-fraction of whey in CRC cell lines focusing on its effects as regulator of SIRT3, mitochondrial metabolic pathways, SIRT3-related metabolic targets regulating the energy metabolism, such as PPAR, LDHA and SREBP, and mitochondrial oxidative metabolism.

## 2. Materials and Methods

### 2.1. Whey Collection and Extraction

Whey of Italian Mediterranean buffalo (*Bubalus bubalis*) milk was provided from commercial buffalo farms located in Southern Italy, as described by [28]. Animals were fed a total mixed ration consisting of maize silage, oat hay, green forage, corn meal and soybean meal and characterized by 0.91 milk forage units (MFU), 15% crude protein on dry matter, and 18% starch, with a forage concentrate ratio of 70:30. The whey was defatted by centrifugation at 5000 rpm for 15 min at 4 °C followed by precipitation of casein by adjusting the pH to 4.6 using hydrochloric acid (1 M). Subsequently, the precipitated casein was removed by centrifugation at 13,000 rpm for 1 h at 4 °C, and the resulting whey fraction was collected in the form of supernatant. In order to recover metabolites with low molecular weight, whey was filtered using 3 kDa cutoff Amicon ultra centrifugal filters and, before being used, was then filtered through 0.22 μm Millipore filters (Merck Millipore, Burlington, MA, USA).

### 2.2. Cell Culture and Treatments

Human colon epithelial CCD 841 CoN cells (CRL-1790), human colorectal adenocarcinoma HCT 116 (CCL-247), HT-29 (HTB-38), SW480 (CCL-228) and LoVo cells (CCL-229) were obtained from American Type Culture Collection (ATCC, Manassas, VA, USA). CCD 841 CoN cells were grown in Eagle’s minimum essential medium (EMEM, 30-2003, ATCC, Manassas, VA, USA), HCT 116 and HT-29 cells were grown in McCoy’s 5A medium (30-2007, ATCC, Manassas, VA, USA). SW480 were maintained in Leibovitz’s L-15 medium (L-15, 30-2008, ATCC, Manassas, VA, USA). LoVo cells were grown in F-12K medium (30-2004, ATCC, Manassas, VA, USA). Cells were grown as a monolayer in a humidified incubator, at 37 °C in a humidified atmosphere with 5% (*v*/*v*) CO_2_, in specific culture medium supplemented with 100 U/mL penicillin, 100 mg/mL streptomycin and 10% fetal bovine serum (FBS, 30-2020, ATCC, Manassas, VA, USA). Medium was changed 2–3 times/week. Cells were seeded into multi-well plates the day before treatments to allow cell attachment. Treatments were performed by culturing cells in complete medium with 3 kDa whey extracts (up to 40% *v*/*v*) up to 72 h, given the high efficiency in inhibiting colon cancer cell viability obtained from 3 kDa milk extracts [6]. Control (Ctr) cells were treated with corresponding volume (% *v*/*v*) of Hanks’ balanced salt solution (HBSS)-10 mM Hepes.

### 2.3. Proliferation Assay

CCD 841 CoN, HCT 116, HT-29, SW480 and LoVo cells were seeded in 96-well plates at the density of 2 × 10^3^ cells/well in the specific serum-free growth medium. Cell proliferation was determined using Cell Counting Kit-8 (CCK-8, Donjindo Molecular Technologies, Inc., Rockville, MD, USA) following manufacturer’s instruction. Briefly, 10 μL of CCK-8 solution was added to each well and cells were incubated at 37 °C for 4 h. Thereafter, absorbance was measured at 450 nm using a microplate reader model 680 Bio-Rad (Bio-Rad, Hercules, CA, USA). Experiments were performed with *n* = 6 replicates. 

### 2.4. Cell Cycle Evaluation

CCD 841 CoN, HCT 116, HT-29, SW480 and LoVo cells (8 × 10^4^ cells/well) were seeded in 6-well plates for treatments. Vital cells were then detached with EDTA-trypsin and stained, without fixing, with BD Cycle test Plus DNA Kit (340242, BD Biosciences, San José, CA, USA) following manufacturer’s instruction. Flow cytometric analysis was performed using a BD Accuri™ C6 (BD Biosciences, San José, CA, USA) by collecting at least 10,000 events. Data analysis was performed with ModFit LT V4.1.7 software. Experiments were performed with *n* = 4 replicates.

### 2.5. Apoptotic Cell Death Assessment

In order to distinguish apoptotic/necrotic from live cells, the FITC Annexin V Apoptosis detection kit (556547, BD Pharmigen, Franklin Lakes, NJ, USA) was used. After trypsinization, cells were washed twice with PBS, resuspended in 500 μL binding buffer 1×, and incubated with 2 μL Annexin V-FITC and 2 μL of the vital dye PI (20 μg/mL) for 30 min. Detection of viable cells, early apoptotic cells, late apoptotic cells, and necrotic cells was performed using a BD Accuri™ C6 (BD Biosciences, San José, CA, USA) cytometer and data analyzed by FlowJo V10 software. For each sample 20,000 events were recorded. Experiments were performed with *n* = 3 replicates.

### 2.6. Detection of Caspase-3/7 Activation

Dual apoptosis assay with NucView 488 caspase-3 substrate and annexin V kit (30,067, Biotium, Fremont, CA, USA) was used for the caspase-3/7 detection and the visualization of apoptotic nuclear morphology, according to the manufacturer’s instruction, using three controls: (1) a negative control with cells not induced to apoptosis; (2) a positive control with cells cultured with 10% *v*/*v* of dimethylsufoxide (DMSO) for 48 h to induce apoptosis, and (3) a caspase-3/7 inhibitor control incubated in cells prior to probe addition. Briefly, cells were seeded in 6-well plates and treated with 40% *v*/*v* whey extract. After trypsinization and a PBS washing, cells were resuspended in 600 μL of 1X Binding Buffer and incubated with 3 μL CF594 Annexin and 3 μL of 0.2 mM NucView 488 Caspase-3 substrate at room temperature for 30 min in the dark. The detection of fluorescence was performed on vital cells with BD Accuri™ C6 and data analyzed by FlowJo V10 software. For each sample 20,000 events were recorded. FACS analysis was carried measuring fluorescence both in FITC (excitation/emission 485/515 nm) and Texas Red (excitation/emission 593/614) channels, proper of each reagent (NucView^®^ 488 caspase-3 substrate and CF^®^594-Annexin V). The analyzed cell population was obtained excluding the threshold, in order to eliminate debris, but it was not selected for physical parameters, so no gating operations were applied. Experiments were performed with *n* = 3 replicates.

### 2.7. Glucose and Lactate Concentration Assays

Intracellular glucose and lactate levels were determined using Glucose Assay Kit-WST (G264, Dojindo Molecular Technologies, Tokyo, Japan) and Lactate Assay Kit-WST (L256, Dojindo, Molecular Technologies, Tokyo, Japan), respectively, according to the manufacturer’s protocol. Absorbance of the colored WST formazan dye was measured at 450 nm using a microplate reader model 680 Bio-Rad (Bio-Rad, Hercules, CA, USA). Glucose and lactate concentrations in each sample were calculated by plotting absorbance values with the calibration curve established with the specific standard, glucose or lactate. Experiments were performed with *n* = 4 replicates.

### 2.8. NAD+/NADH Ratio Evaluation

NAD+/NADH ratio was measured using NAD/NADH Assay Kit-WST (N509, Dojindo Molecular Technologies, Tokyo, Japan) according to manufacturer’s protocols. HCT 116, HT-29, SW480 and LoVo cells were seeded in 6-well plates and treated for 48 and 72 h with 40 % *v*/*v* whey. For each cell line, a cell suspension (5.0 × 10^5^ cells) was prepared, washed with PBS and treated with NAD+/NADH extraction buffer. The sample solution for the amount of NADH was incubated at 60 °C for 1 h, for decomposing NAD+ content in the sample. After incubation, the samples, total NAD+/NADH, and standard series were treated with working reagent and incubated at 37 °C for 1 h. The absorbance at 450 nm was measured by using a microplate reader model 680 Bio-Rad (Bio-Rad, Hercules, CA, USA) and the amount of total NAD+/NADH and NADH in the samples was estimated using a calibration curve. The amount of NAD+ was calculated by subtracting the amount of NADH from the total NAD+/NADH. Experiments were performed with *n* = 3 replicates.

### 2.9. GSH/GSSG Ratio Assessment

The ratio of GSH and GSSG, as index of oxidative stress, was determined using a GSSG/GSH quantification kit (G257, Dojindo Molecular Technologies, Tokyo, Japan) according to the supplier’s instructions. Cells were seeded in 6-well plates and treated for 48 and 72 h with 40 % *v*/*v* whey extracts or with 20 µM glutathione S-transferase inhibitor (ethacrynic acid, ab141399, Abcam, Cambridge, UK) or inducer (dihydromyristicin, ab142290, Abcam, Cambridge, UK). Cells were scraped, washed with PBS and two sets of sample solutions (200 μL each) were prepared to determine GSH and GSSG levels. In addition, GSH and GSSG standard solutions, were also assembled. After 1 h incubation at 37 °C substrate and enzyme/coenzyme working solutions were added to each well. After incubation for 10 min at 37 °C, the absorbance at 405 nm was measured by using a microplate reader model 680 Bio-Rad (Bio-Rad, Hercules, CA, USA). The concentrations of GSH and GSSG in the samples were calculated using their proper calibration curves. All experiments were performed with *n* = 3 replicates. 

### 2.10. SIRT3 Fluorescence Assay

SIRT3 Fluorometric drug discovery assay (BML-AK557, Enzo Life, Farmingdale, NY, USA) was performed following manufacturer’s instruction. Briefly, 50 μL of flour de lys substrate and NAD+, sirtuin substrate, was added in each well and incubated for 5 h at 37 °C. In order to stop SIRT3 activity and to produce the fluorescent signal, 50 µL of Developer II and 2 mM of nicotinamide (NAM), sirtuin inhibitor, was added. After incubation for 30 min at 37 °C, the fluorescence was measured at an excitation wavelength of 360 nm and at an emission light of 460 nm using Tecan Infinite 2000 Multiplate reader.

### 2.11. SIRT3 Silencing

The SIRT3 gene was transiently silenced in human colorectal adenocarcinoma HCT 116 and HT-29 cells using SIRT3 siRNA Oligos set for specific human Sirtuin 3 (438080910101, Applied Biological Materials, Inc., Richmond, BC, Canada), according to the manufacturer’s instructions. Briefly, HCT 116 and HT-29 cells were seeded in 6-well plate in complete culture medium. After 24 h, the growth medium was removed and cells were transfected with control non-targeting siRNA (NT-siRNA) (50 nM) and with small interfering RNA (siRNA) (50 nM), consisted of a mixture of three target sequences for SIRT3, in serum- and antibiotic-free medium. The silencing was performed using Lipofectamin as transfection reagent. Cells were incubated for 8 h, followed by additional 12 h of incubation after the addition of FBS (10%) directly to each well. Transfected cells were then treated with whey extracts.

### 2.12. Evaluation of SIRT3 mRNA Levels

Total RNA was extracted from treated cells using the Total RNA purification kit (17200, Norgen Biotek Corp., Thorold, ON, Canada), following the manufacturer’s protocol, and then quantified using a NanoDrop2000 spectrophotometer (Thermo Fisher Scientific, Waltham, MA, USA). For each sample, 400 ng of the total RNA was reverse transcribed to cDNA and this amplified using MyTaq™ One-Step RT-PCR kit (BIO-65049, Meridian Bioscience Inc., Cincinnati, OH, USA), according to manufacturer’s instruction. The PCR reactions were performed on a thermal cycler SureCycler 8800 (Agilent Technologies, Santa Clara, CA, USA). Total reaction volume was 25 µL. The reverse transcription was performed at 45 °C for 20 min while the amplification program included a preincubation step for polymerase activation (1 min, 95 °C), followed by 40 cycles consisting of a denaturation step (10 s, 95 °C), an annealing step (10 s, 62 °C), and an elongation step (30 s, 72 °C). The oligonucleotide primers for human SIRT3 and glyceraldehyde-3-phosphate dehydrogenase (GAPDH) were as follows: SIRT3-F 5′-CGGCTCTACACGCAGAACATC-3′; SIRT3-R 5′-CAGCGGCTCCCCAAAGAACAC-3′; GAPDH-F5′-AACGGGAAGCTTGTCATCAA-3′; GAPDH-R 5′-TGGACTCCACGACGTACTCA-3′. For relative quantification, all samples were normalized using GAPDH as an internal reference gene. A negative control lacking cDNA template was run in each assay.

### 2.13. Cell Lysis and Western Blotting Analysis

Cells were lysed with RIPA lysis buffer (1% NP-40, 0.5% sodium deoxycholate, 0.1% SDS in PBS) containing 10 μg/mL aprotinin, leupeptin and 1 mM phenylmethylsulfonyl fluoride (PMSF). After incubation on ice for 1 h, the cell lysates were centrifuged at 10,000× *g* for 15 min at 4 °C and protein content was determined by using Bio-Rad Protein Assay kit (Bio-Rad, Hercules, CA, USA) and compared with a bovine serum albumin (BSA) standard curve. Protein samples (20–50 μg/lane) were resolved by SDS-PAGE and proteins were then transferred to nitrocellulose membranes (Bio-Rad, Hercules, CA, USA). Non-specific binding sites were blocked using 1% BSA in phosphate-buffered saline (pH 7.4) containing 0.1% Tween-20 (PBS-T) for 1 h at 25 °C. The blocking buffer was drained and the membrane was allowed to incubate in primary antibodies diluted in blocking buffer overnight at 4 °C: anti-SIRT3 (1:2000, PA5-86035, Invitrogen Corporation, Waltham, MA, USA), anti-sterol regulatory element-binding protein 1 (SREBP1, 1:1000, Abcam, ab28481, Cambridge, UK), anti-peroxisome proliferator-activated receptor α (PPAR-α, 1:1000, Elabscience Biotechnology Inc., Houston, TX, USA, E-AB-32646), anti-PPAR-γ (1:1000, Biorbyt, orb69095, Cambridge, UK), anti-lactate dehydrogenase A (LDHA, 1:1000, ThermoFisher Scientific, PA5-27406, Waltham, MA, USA), anti-cyclin B1 (1:1000, Cell Signaling Technology, 4138), anti-cyclin D1 (1:1000, Abcam, Cambridge, UK, ab134175), anti-α-tubulin (1:5000, Elabscience Biotechnology Inc., Houston, TX, USA, E-AB-20036), anti-γ-tubulin (1:2000, Sigma-Aldrich, St. Louis, MO, USA, T6557), anti-glyceraldehyde-3-phosphate dehydrogenase (GAPDH, 1:2000, ab9485, Abcam, Cambridge, UK) and anti-β-actin (1:5000, Abcam, Cambridge, UK, ab8227). After incubation for 1 h with HRP-conjugated secondary antibodies (GxMu-003-DHRPX and GtxRb-003-DHRPX, ImmunoReagents Inc., Raleigh, NC, USA), the immunocomplexes were examined on dried nitrocellulose membranes by Excellent chemiluminescent sustrate kit (E-IR-R301, Elabscience Biotechnology Inc., Houston, TX, USA) and visualized using ChemiDoc Imaging System with Image Lab 6.0.1 software (Bio-Rad Laboratories). After background subtraction, the densities of immunoreactive bands were measured with ImageJ software (National Institutes of Health) and expressed as arbitrary units (AU).

### 2.14. Statistical Analysis

All experiments were performed in at least three replicates and reported data expressed as mean ± standard deviation (SD). Statistical analysis between two groups was performed using Student’s t test while the differences among three groups were analyzed by one-way ANOVA followed by Tukey post hoc test. Each difference with *p* < 0.05 was considered statistically significant.

## 3. Results

### 3.1. Inhibition of Proliferation

**Figure 1:**
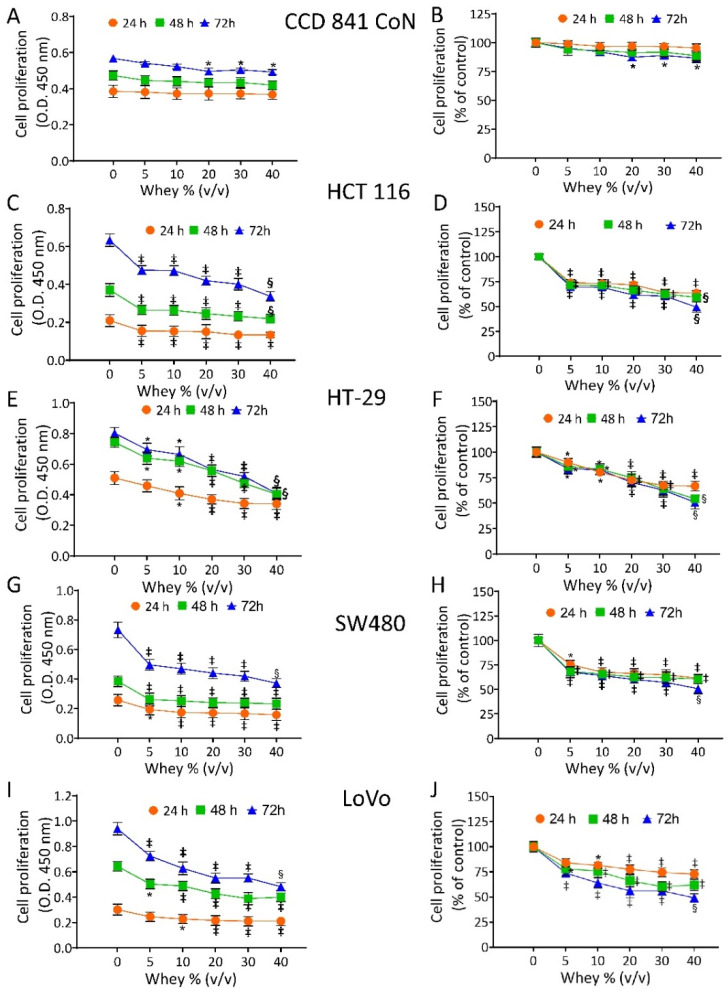
Whey specifically inhibits CRC cell proliferation. Cell proliferation was evaluated in (**A**,**B**) CCD 841 CoN, (**C**,**D**) HCT 116, (**E**,**F**) HT-29, (**G**,**H**) SW480 and (**I**,**J**) LoVo cell lines after exposure to different volumes of whey (0, 5, 10, 15, 20 and 40% *v*/*v*) for different times (24, 48 and 72 h). Cell proliferation was assessed using Cell Counting Kit-8 assay (Donjindo Molecular Technologies, Tokyo, Japan) and reported as optical density (O.D.) values and as percentage of control. Control cells (0% *v*/*v*) were grown in medium containing 40% *v*/*v* of HBSS-10 mM Hepes. Values represent the mean ± SD of three independent experiments. * *p* < 0.05 vs. Ctr; ‡ *p* < 0.01 vs. Ctr; § *p* < 0.001 vs. Ctr.

The effect of whey on cell proliferation was assessed on non-malignant cells (CCD 841 CoN) and CRC cell lines (HT-29, HCT 116, LoVo and SW480). Cell viability of CCD 841 CoN was not affected at 24 h and 48 h. The treatment for 72 h only minimally affected the viability of the normal cells, as 81.3% ± 0.70 proliferation was observable at the highest volume of whey tested (40% *v*/*v*) (Figure 1A,B).

In CRC cells, whey inhibited proliferation in a time- and dose-dependent manner. HCT 116 cells reached the IC50 at 72 h with 40 % *v*/*v* of whey (*p* < 0.001 vs. Ctr) (Figure 1C,D). HT-29 cells displayed a lower proliferative percentage (49.0% ± 1.39, *p* < 0.001 vs. Ctr) (Figure 1E,F) and SW480 cells showed the highest inhibition value of 52.1% ± 2.04 (*p* < 0.001 vs. Ctr) of residual proliferation (Figure 1G,H). Finally, LoVo cells maintained 51.3% ± 1.11 (*p* < 0.001 vs. Ctr) proliferation after 72 h with 40 % *v*/*v* of whey (Figure 1I,J). No cytotoxic effects were reported when cell lines were incubated up to 72 h with increasing volumes of HBSS-10 mM Hepes (Supplementary Figure S1). Based on these results, further studies were performed by incubating CRC cell lines with 40 % *v*/*v* whey for different times.

### 3.2. Cell Cycle Perturbation

**Figure 2:**
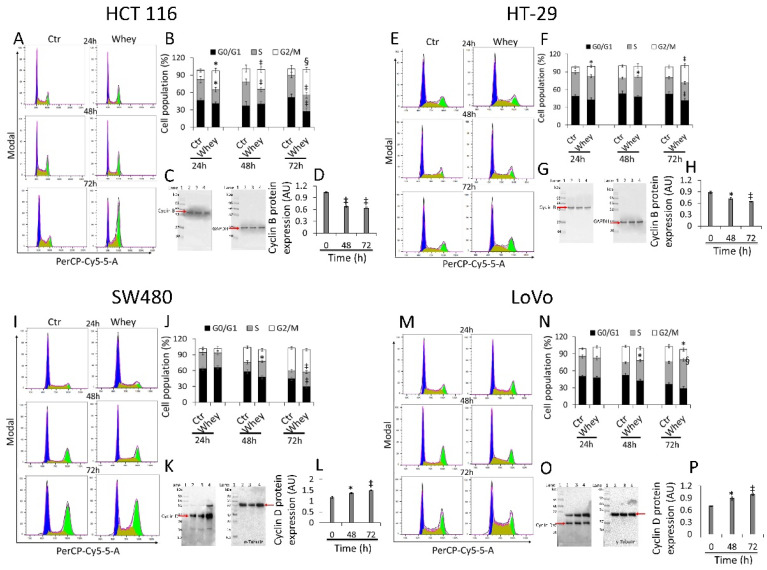
Whey regulates cell cycle in CRC cells. Cell lines were treated with whey 40% *v*/*v* up to 72 h. Representative cell cycle analysis and full-length blots of Western blotting examination of cyclin B in (**A**–**D**) HCT 116 and (**E**–**H**) HT-29 cells or cyclin D in (**I**–**L**) SW480 and (**M**–**P**) LoVo cells. Cell distribution was assessed by flow cytometry collecting PI fluorescence as FL3-A (linear scale). For each sample at least 10,000 events were collected and analysis performed by using ModFIT software. Control (Ctr) cells were treated with corresponding volume (40% *v*/*v*) of HBSS-10 mM Hepes. Lane 1 = molecular markers; Lane 2 = 0 h; lane 3 = 48 h; lane 4 = 72 h. Protein expression was determined, after normalization with internal control (α- or γ-tubulin), with ImageJ software and values expressed as arbitrary units (AU). * *p* < 0.05 vs. Ctr or 0 h; ‡ *p* < 0.01 vs. Ctr or 0 h; § *p* < 0.001 vs. Ctr.

HCT 116 cells treated with whey (40% *v*/*v*) for different times showed a time-dependent arrest in G2/M phase, more consistent after 48 and 72 h (33.9 ± 4.87 and 43.7% ± 4.21, respectively, vs. 11.3% ± 5.07 in Ctr, *p* < 0.001) (Figure 2A,B) and a time-dependent decrease of cyclin B (*p* < 0.01) (Figure 2C,D and Figure S2).

Similarly, HT-29 cell cycle analysis showed a block in G2/M phase, more consistent after 48 and 72 h of incubation with whey (16.6 ± 2.23 and 29.0% ± 4.01, respectively, vs. 9.03% ± 1.69 in Ctr, *p* < 0.01) (Figure 2E,F). As for HCT 116, downregulation of cyclin B expression levels was also detected (*p* < 0.01) (Figure 2G,H and Figure S2). On the other hand, SW480 showed an accumulation in S phase after 48 and 72 h (27.4 ± 3.56 and 30.1% ± 4.51, respectively, vs. 14.5% ± 2.78 in Ctr, *p* < 0.01) (Figure 2I,J) and a time-dependent accumulation of protein cyclin D (*p* < 0.01) (Figure 2K,L and Figure S2). Likewise, also the metastatic CRC cell line, LoVo, showed a consistent S phase accumulation after 48 and 72 h (35.5 ± 2.97 and 50.9% ± 4.25, respectively, vs. 22.3% ± 2.85 in Ctr, *p* < 0.001) (Figure 2M,N) and upregulation of cyclin D protein (*p* < 0.05) (Figure 2O,P and Figure S2). Whey treatment (40% *v*/*v*) up to 72 h did not perturbate the cell cycle in normal colon CCD 841 CoN cells (Figure S3), supporting the specificity of the effect for cancer cells.

### 3.3. Induction of Apoptosis

**Figure 3:**
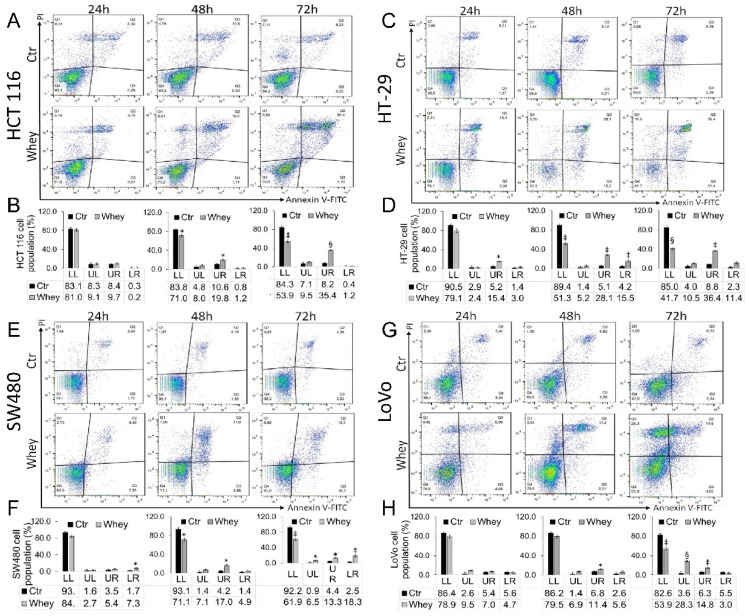
Whey induces apoptosis in CRC cells. Representative dot plots and analyses of Annexin V-FITC and Propidium Iodide (PI)-stained (**A**,**B**) HCT 116, (**C**,**D**) HT-29, (**E**,**F**) SW480 and (**G**,**H**) LoVo cells. Cell lines were treated with HBSS-10 mM Hepes (Ctr) or whey 40% *v*/*v* for 24, 48 and 72 h and cell viability/death assessed by flow cytometry. Lower left (LL), viable cells; upper left (UL), necrotic cells; lower right (LR), early apoptotic cells; upper right (UR), late apoptotic cells. Data are expressed as mean ± SD of *n* = 3 experiments. At least 10,000 events were acquired. * *p* < 0.05 vs. Ctr; ‡ *p* < 0.01 vs. Ctr; § *p* < 0.001 vs. Ctr.

Treatment up to 72 h with whey (40% *v*/*v*) induced a time-dependent increase of late apoptotic population in HCT 116 cells (*p* < 0.001) (Figure 3A,B), an increase of necrosis and apoptosis (both early and late) in HT-29 cells (*p* < 0.001) (Figure 3C,D) and SW480 cells (*p* < 0.01) (Figure 3E,F). In LoVo cells, the necrosis and late apoptosis induced by whey reached the highest values after 72 h treatment (*p* < 0.001) (Figure 3G,H). Whey weakly induced an apoptotic cell death in normal colon CCD 841 CoN cells after 72 h treatment (Figure S4).

### 3.4. Caspase-3 Activation

**Figure 4:**
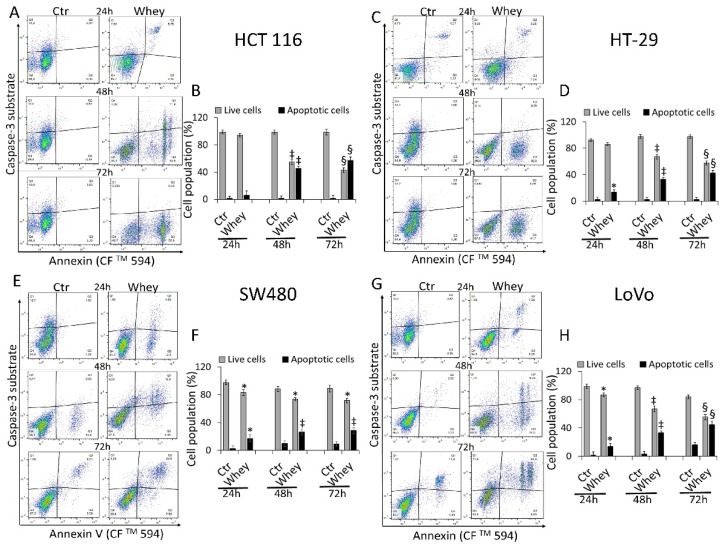
Whey mediates caspase-3 activation in CRC cells. Representative dot plots and analyses of caspase-3 activation in (**A**,**B**) HCT 116, (**C**,**D**) HT-29, (**E**,**F**) SW480 and (**G**,**H**) LoVo cells. Cells were treated with HBSS-10 mM Hepes (Ctr) or whey 40% *v*/*v* for 24, 48 and 72 h and cell population assessed by flow cytometry. Data are expressed as % of live or apoptotic population with mean ± SD of *n* = 3 experiments. At least 10,000 events were acquired. * *p* < 0.05 vs. Ctr; ‡ *p* < 0.01 vs. Ctr; § *p* < 0.001 vs. Ctr.

The levels of activated caspase-3 were then analyzed by flow cytometry and reported as apoptotic cells (%). HCT 116 cells displayed a time-dependent activation of caspase-3 gaining the maximum activity rate at 72 h (57.3% ± 1.2 of apoptotic cells vs. 1.2% ± 0.21 in Ctr, *p* < 0.001) (Figure 4A,B).

HT-29 treated with whey showed an increase of caspase-3 activation up to 72 h (42.6% ± 3.1 of apoptotic cells vs. 2.4% ± 0.3 in Ctr, *p* < 0.001) (Figure 4C,D). In SW480 cells, caspase-3 was activated to a lower extent even after 72 h of incubation with whey (28.4% ± 2.8 of apoptotic cells vs. 9.1% ± 1.1 in Ctr, *p* < 0.01) (Figure 4E,F). Finally, the whey dependent capability to induce apoptotic cell death through caspase-3 activation after 72 h was also observed in LoVo cells (44.8% ± 2.4 vs. 16.2% ± 2.2 in Ctr, *p* < 0.001) (Figure 4G,H). Since the effects of whey on caspase 3 activation were most marked after 48 and 72 h, further experiments were conducted using these two incubation times. No effects were observed in CCD 841 CoN cells after 72 h of whey treatment (Figure S5).

### 3.5. Redox State and Energy Metabolism Modulation 

**Figure 5:**
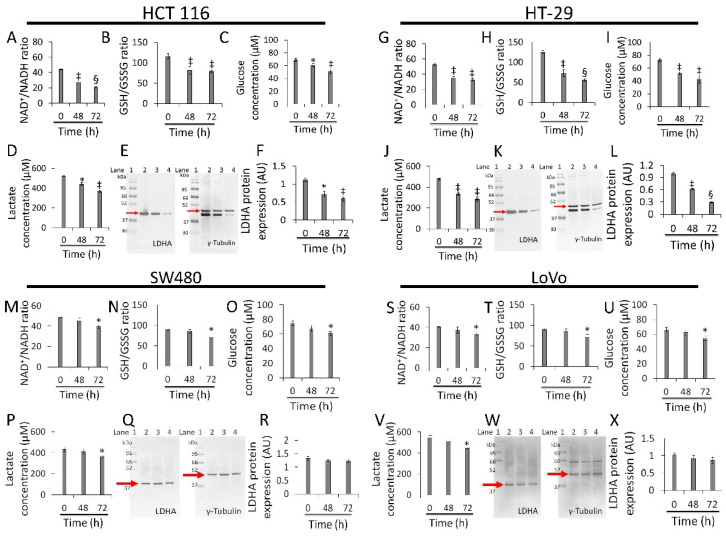
Whey affects energy metabolism in CRC cells. The effects of whey on NAD+/NADH, GSH/GSSG, glucose uptake, lactate production, and LDHA protein expression levels were detected on (**A**–**F**) HCT 116, (**G**–**L**) HT-29, (**M**–**R**) SW480 and (**S**–**X**) LoVo cells. Cells were treated with whey 40% *v*/*v*. Lane 1 = molecular markers; Lane 2 = 0 h; lane 3 = 48 h; lane 4 = 72 h. LDHA protein expression was determined, after normalization with γ-tubulin as internal control, with ImageJ software and values expressed as arbitrary units (AU). * *p* < 0.05 vs. 0 h; ‡ *p* < 0.01 vs. 0 h; § *p* < 0.001 vs. 0 h.

Whey strongly inhibited NAD+/NADH and GSH/GSSG ratios, glucose uptake, lactate production, and LDHA expression levels in HCT 116 (*p* < 0.01 vs. 0 h) (Figure 5A–F and Figure S6) and HT-29 cells (*p* < 0.001 vs. 0 h) (Figure 5G–L Figure S6). Less pronounced effects were observed in SW480 (*p* < 0.05 vs. 0 h) (Figure 5M–R Figure S6) and LoVo cells (*p* < 0.05 vs. 0 h) (Figure 5S–X Figure S6). Positive and negative controls were also tested for the study of redox state (Figure S7).

### 3.6. Regulation of SIRT3 Expression and Activity 

**Figure 6:**
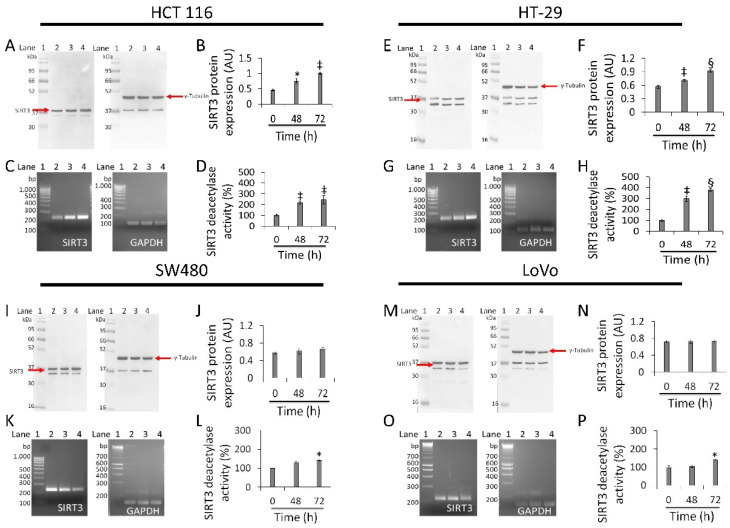
Antiproliferative effects of whey are mediated by SIRT3 modulation. SIRT3 protein, mRNA levels and enzyme activity were assessed on (**A**–**D**) HCT 116, (**E**–**H**) HT-29, (**I**–**L**) SW480 and (**M**–**P**) LoVo cells treated with whey (40% *v*/*v*). Lane 1 = molecular markers; Lane 2 = 0 h; lane 3 = 48 h; lane 4 = 72 h. SIRT3 protein expression was normalized against γ-tubulin and determined with ImageJ software and values expressed as arbitrary units (AU). For PCR products, SIRT3 mRNA levels were normalized against GAPDH internal control and the amplified transcripts are shown with the expected sizes on 2.0% agarose gels.* *p* < 0.05 vs. 0 h; ‡ *p* < 0.01 vs. 0 h; § *p* < 0.001 vs. 0 h.

In HCT 116 cells, whey induced a time-dependent upregulation of SIRT3 protein (1.0 ± 0.02 AU vs. 0.45 ± 0.03 AU, *p* < 0.01), enzyme activity (244.5 ± 30.3 vs. 100% of 0 h, *p* < 0.01), and mRNA (Figure 6A–D and Figure S8). HT-29 cells also showed upregulated levels of SIRT3 protein (0.92 ± 0.02 AU vs. 0.56 ± 0.03 AU, *p* < 0.001), mRNA levels and enzyme activity (380.5 ± 11.8 vs. 100%, *p* < 0.001) (Figure 6E–H and Figure S8). Conversely, in SW480 cells treatment with whey did not enhance neither SIRT3 protein (Figure 6I,J and Figure S8) nor its mRNA levels (Figure 6K). However, a weak rise in deacetylase activity was observed (*p* < 0.05 vs. 0 h) (Figure 6L). Likewise, in LoVo cells, whey did not show any effect on SIRT3 (Figure 6M–O and Figure S8) with an increase only in its enzymatic activity (*p* < 0.05 vs. 0 h) (Figure 6P).

### 3.7. Mitochondrial Metabolic Pathway

**Figure 7:**
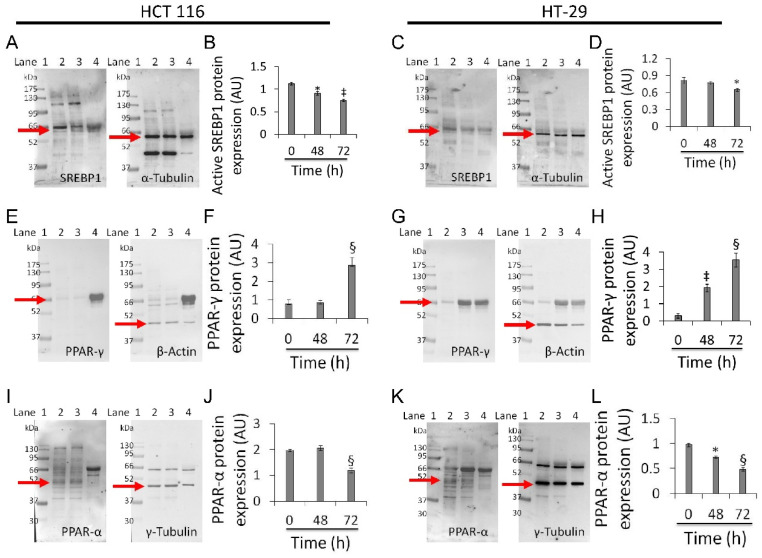
Whey modulates mitochondrial metabolic markers in CRC cells. (**A**–**D**) Active SREBP1, (**E**–**H**) PPAR-γ and (**I**–**L**) PPAR-α protein levels in HCT 116 and HT-29 were detected by western blotting analysis after treatment with whey (40% *v*/*v*) up to 72 h. Lane 1 = molecular markers; Lane 2 = 0 h; lane 3 = 48 h; lane 4 = 72 h. The mitochondrial marker levels were normalized with internal controls (β-actin, α- or γ-tubulin) with ImageJ software and values expressed as arbitrary units (AU). * *p* < 0.05 vs. 0 h; ‡ *p* < 0.01 vs. 0 h; § *p* < 0.001 vs. 0 h.

Based on the above results, experiments aimed at investigating the effect of whey on sterol regulatory-element binding protein 1 (SREBP1), peroxisome proliferator-activated receptor (PPAR)-γ, and PPAR-α were performed on the two most responsive CRC cell lines, HCT 116 and HT-29 (Figure 7).

Whey treatment decreased levels of the active form of SREBP1 in HCT 116 (0.75 ± 0.02 AU vs. 1.1 ± 0.03 AU, *p* < 0.01) and HT-29 (0.65 ± 0.02 AU vs. 0.82 ± 0.05 AU, *p* < 0.05) (Figure 7A–D and Figure S9), and increased PPAR-γ expression (2.8 ± 0.4 AU vs. 0.77 ± 0.2 AU and 3.5 ± 0.4 AU vs. 0.30 ± 0.1, respectively, *p* < 0.001) (Figure 7E–H and Figure S9). Both cell lines also showed a decreased expression of PPAR-α protein after 72 h exposure with whey (*p* < 0.001 vs. 0 h) (Figure 7I–L and Figure S9).

### 3.8. SIRT3 Silencing

**Figure 8:**
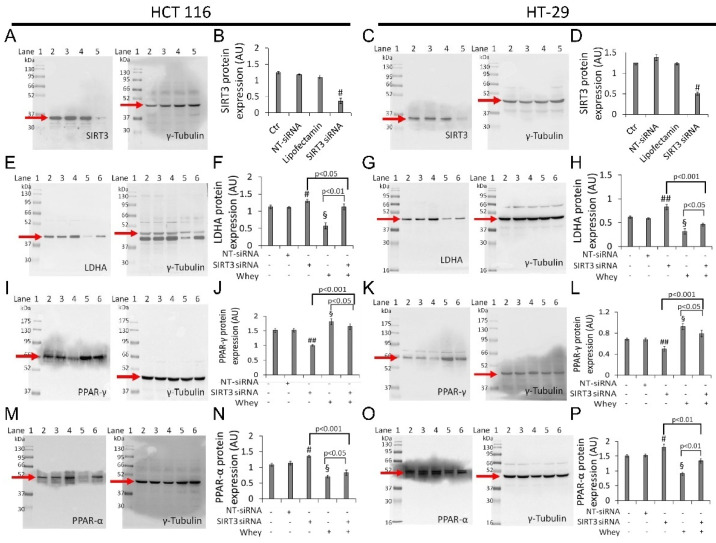
Whey effects in HCT 116 and HT-29 cells occur via SIRT3. Cells were transfected with scramble siRNA (NT-siRNA), transfection reagent (Lipofectamin) and SIRT3 siRNA, as described under Methods section. Protein expression levels of SIRT3 in (**A**,**B**) HCT 116 and (**C**,**D**) HT-29. Lane 1 = molecular markers; Lane 2 = Ctr; lane 3 = NT-siRNA; lane 4 = Lipofectamin; Lane 5 = SIRT3 siRNA. § *p* < 0.001 vs. Ctr. After SIRT3 silencing, HCT 116 and HT-29 cells were treated with whey (40% *v*/*v*) for 72 h and (**E**–**H**) LDHA, (**I**–**L**) PPAR-γ, and (**M**,**P**) PPAR-α expression levels were determined. Lane 1 = molecular markers; Lane 2 = Ctr; lane 3 = NT-siRNA; lane 4 = SIRT3 siRNA; lane 5 = whey 40% *v*/*v* for 72 h; lane 6 = SIRT3 siRNA + whey 40% *v*/*v* for 72 h. The protein levels were normalized using γ-tubulin as internal control with Image J software and values expressed as arbitrary units (AU). § *p* < 0.001 vs. Ctr; # *p* < 0.05 vs. NT-siRNA; ## *p* < 0.01 vs. NT-siRNA.

Transient SIRT3 gene silencing with small interfering RNA (SIRT3 siRNA), (Supplementary Table S1) as confirmed by the decreased protein levels (*p* < 0.001 vs. Ctr) (Figure 8A–D and Figure S10), supported the key role of SIRT3 in mediating the cytotoxicity and the metabolic reprogramming induced by whey in HCT 116 and HT-29 cells (SIRT3 siRNA + whey). 

In detail, silenced HCT 116 and HT-29 cells (SIRT3 siRNA) showed upregulation of LDHA (1.30 ± 0.5 vs. 1.10 ± 0.3 in HCT 116, *p* < 0.05; 0.83 ± 0.05 vs. 0.58 ± 0.02 in HT-29, *p* < 0.001) (Figure 8E–H and Figure S10) and PPAR-α protein levels (1.35 ± 0.04 vs. 1.13 ± 0.06 in HCT 116, *p* < 0.05; 1.80 ± 0.10 AU vs. 1.52 ± 0.05 AU in HT-29, *p* < 0.05) (Figure 8M–P and Figure S10), and downregulation of PPAR-γ (1.0 ± 0.03 AU vs. 1.53 ± 0.07 in HCT 116, *p* < 0.01; 0.50 ± 0.04 vs. 0.67 ± 0.03 in HT-29, *p* < 0.05) (Figure 8I–L and Figure S10).

## 4. Discussion

**Figure 9:**
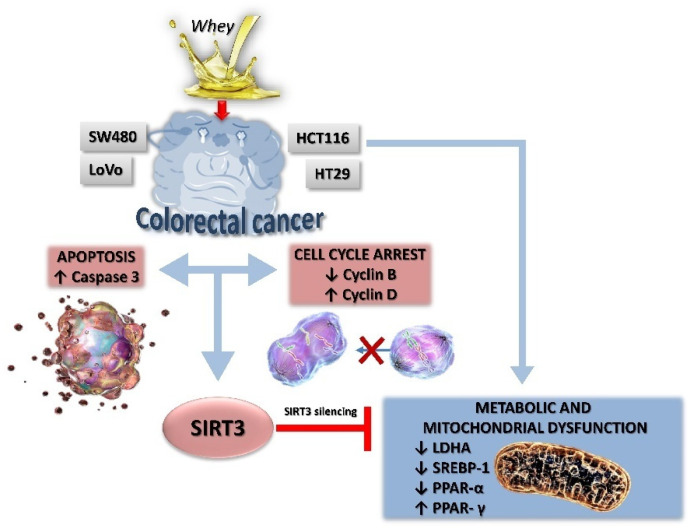
A proposed schematic representation of the effects of whey on SIRT3 and metabolic reprogramming in human CRC cells. Metabolic reprogramming and mitochondrial dysfunction induced by whey were blocked by SIRT3 gene silencing, suggesting a key role of this sirtuin in the antiproliferative effects of whey.

In this study, we provided evidence on the in vitro anticancer activity of whey on CRC cells by modulating SIRT3 and metabolic reprogramming. Whey treatment displayed cytotoxic, apoptotic and cycle modulating properties on LoVo, SW480, HCT 116 and HT-29 cells with higher effects on HCT 116 and HT-29 cells, reaching 50% of cell death. Of interest, whey acted differently on the distinct cell lines, although promoting antineoplastic effects. Indeed, in HT-29 and HCT 116 cells, whey determined a negative modulation of cyclin B, causing a G2/M phase accumulation, whereas in LoVo and SW480 cells upregulation of cyclin D, and G1/S cell cycle arrest were observed. Apoptosis occurred in all cell lines starting from 48 h of treatment with the highest efficiency observed in HCT 116 and HT-29 after 72 h of incubation. Moreover, LoVo and SW480 whey-treated cells undergone to necrosis process (Figure 9).

Cancer cells show increased glucose uptake and lactate production, indicating defects in mitochondrial respiration [36]. In this contest, disruption of mitochondrial respiration, in a way and extent that cannot be counterbalanced by cancer cells, might counteract the malignant process, independently of its origin. We found that whey was active in inducing metabolic dysfunction in CRC cells, leading to a decreased glucose uptake, lactate production, NAD+/NADH and GSH/GSSG ratio. Previous studies reported that supplementation with whey protein concentrate selectively depletes tumor GSH levels [32].The reduction of GSH levels has been shown to regulate both extrinsic and intrinsic apoptotic signaling cascades at different checkpoints [37]. The in vitro protective effects of whey occur also through a negative regulation of SREBP1 whose elevated activity has been implicated in the tumorigenesis of CRC [38,39]. Indeed, SREBP1 gene silencing has been shown to inhibit CRC cell proliferation, migration, and invasion and promote apoptosis [38,39].

The bioenergetic signature in CRC includes increased expression of glycolytic and repressed mitochondrial respiration [40] with decreased mitochondrial bioenergetic activity in the aggressive clones of CRC cells which, in turn, determines repression of genes involved in mitochondrial biogenesis and function [40]. Mitochondrial function, including metabolism, ATP generation and the oxidative stress response, is critically regulated by SIRT3 [24], the major deacetylase within the mitochondrial matrix acting as a tumor suppressor by inhibiting the Warburg effect [41,42]. SIRT3 orchestrates multiple pathways and acts differently in various types of cancer cells, as tumor suppressor but also as tumor promoter [18,19,20,21,22,23,24,25,26]. Intriguingly, in CRC, SIRT3 displays an oncogenic role by deacetylating SHMT2 [27], and acts as tumor suppressor by increasing ROS production and PINK1/Parkin/mitophagy axis activation [9]. Results of this study indicated that whey treatment increased the SIRT3 expression levels in HT-29 and HCT 116 cells. The role of SIRT3 in cellular energetics and tumorigenesis has been extensively studied, as it mediates metabolic reprogramming by regulating the expression of a broad range of gene, including PPAR-α, PPAR-γ, and PPAR-δ. To date, the exact role of PPAR as CRC suppressors or promoters is not straightforward, as there is no clear unifying accepted mechanism explaining the role of PPAR on promotion/development of colon cancer. The high expression of PPAR-α in pancreatic cancer patients and its association with adverse prognosis provided robust evidence for the oncogenic effect of this receptor [43]. PPAR-α is activated by nutrients and influences the transcriptional activity of the oncogene Src, which in turn, results in CRC cell invasion and metastasis [44]. PPAR-γ plays critical roles in lipid storage, glucose metabolism, energy homeostasis, inflammation, and cancer. Its activation in colon carcinogenesis has largely been debated, thus inducing apoptosis and preventing cancer cell proliferation, development, and reducing tumor inflammatory microenvironment. While some evidence supports the cancer promoting effects of PPAR-γ in CRC, other studies suggest that it may act in an opposite direction, impeding its development and progression [45,46]. A recent study, performed on 100 primary tumor tissues and normal adjacent tissues from CRC patients provided the relationship between PPAR expression levels and progression or worse prognostic effects of CRC [34]. A significant increase in the expression of PPAR-α in the CRC tissues was associated with advanced tumor, node and metastasis stage, grade, and size. In contrast, an inverse correlation was found between PPAR-γ expression and the above mentioned clinicopathological factors [34], indicating that PPAR-γ can act as a tumor suppressor in CRC. These observations supported our findings showing that the growth inhibition and apoptosis induced by whey are linked to the downregulation of PPAR-α and the positive modulation of PPAR-γ. SIRT3 silencing by small interfering RNA opposed the effect of whey on PPAR-γ, PPAR-α, and LDHA suggesting that modulation of mitochondrial dysfunction and metabolism is directly/indirectly mediated by SIRT3. Although whey did not show the same effect on all cell lines used in this study, a different responsiveness to it was observed between the cells that mimic a primary colon adenocarcinoma, HCT 116 and HT-29, and the most aggressive forms bearing from the adjacent environment and a metastatic site (Dukes’ type B and D), SW480 and LoVo. These features could explain the different CRC cell susceptibility to whey treatment. Indeed, differences in tumor microenvironment might translate into distinct responsiveness to cell cycle changes and apoptosis.

The mechanism(s) by which nutrients may affect health span and act as nutritional epigenetic modulators is still not fully understood as it involves multiple evolutionary conserved nutrient-sensing pathways interacting each other, such as sirtuins, conserved key regulators mammalian target of rapamycin, AMP-activated protein kinase, insulin/insulin-like growth factor 1, PINK1/Parkin, and mitophagy [1,2,9]. It is undoubtedly hard to define the exact bioactive component of the 3 kDa whey extract, known to be rich in peptides, betaines, l-carnitine, and acylcarnitines [28], especially in light of the synergism among betaines [8]. Moreover, in order to outline the precise effect of whey and unveil other upregulated proteins in addition to SIRT3, genome-wide analysis applied to in vivo studies, as well as to a largest panel of cancer cell lines overexpressing SIRT3, will deepen the knowledge on the molecular mechanism through which SIRT3 mediates the antitumor effects of whey in CRC cells.

## 5. Conclusions

Altogether, findings of this study indicate that 3 kDa whey extracts showed the same cytotoxic and apoptotic activities of milk in CRC cells and added knowledge on the mechanism of action that involves the modulation of SIRT3 and mitochondrial metabolism. Finally, in addition to the relevance of the health effects of food nutrients, the potential of using a by-product of the dairy industry to proceed along an environmental sustainability for generations to come should not be overlooked.

## 6. Patents

Patent n. 102021000024911; deposition date, 29 September 2021.

## Data Availability

The data presented in this study are available from the corresponding author upon request.

## Supplementary Materials

The following are available online at https://www.mdpi.com/article/10.3390/cancers13205196/s1. Figure S1: Whey effects on colorectal cell proliferation, Figure S2: Image representing untouched, uncropped gels related to Figure 2, Figure S3: Whey treatment does not modulate the CCD 841 CoN colon cell cycle, Figure S4: Whey does not display apoptotic effects in CCD 841 CoN cells, Figure S5: Whey does not mediate caspase-3 activation in non-tumoral colon cells, Figure S6: Image reporting untouched, uncropped gels related to Figure 5, Figure S7: Redox state controls, Figure S8: Image reporting untouched, uncropped gels related to Figure 6, Figure S9: Image reporting untouched, uncropped gels related to Figure 7, Figure S10: Image reporting untouched, uncropped gels related to Figure 8, Table S1: Oligo sequences for SIRT3 silencing and negative control (NT-siRNA).
