# Supplementary material for: SIRT3 and Metabolic Reprogramming Mediate the Antiproliferative Effects of Whey in Human Colon Cancer Cells
Source: Cancers (Basel). 2021 Oct 16;13(20):5196. doi: 10.3390/cancers13205196 (PMC8533739; doi:10.3390/cancers13205196)
Supplement: Supplementary file 1 [file cancers-13-05196-s001.zip › cancers-1426474-SI.pdf]

SIRT3 and metabolic reprogramming mediate the antiproliferative effects of whey in human colon cancer cells

SUPPLEMENTARY MATERIALS

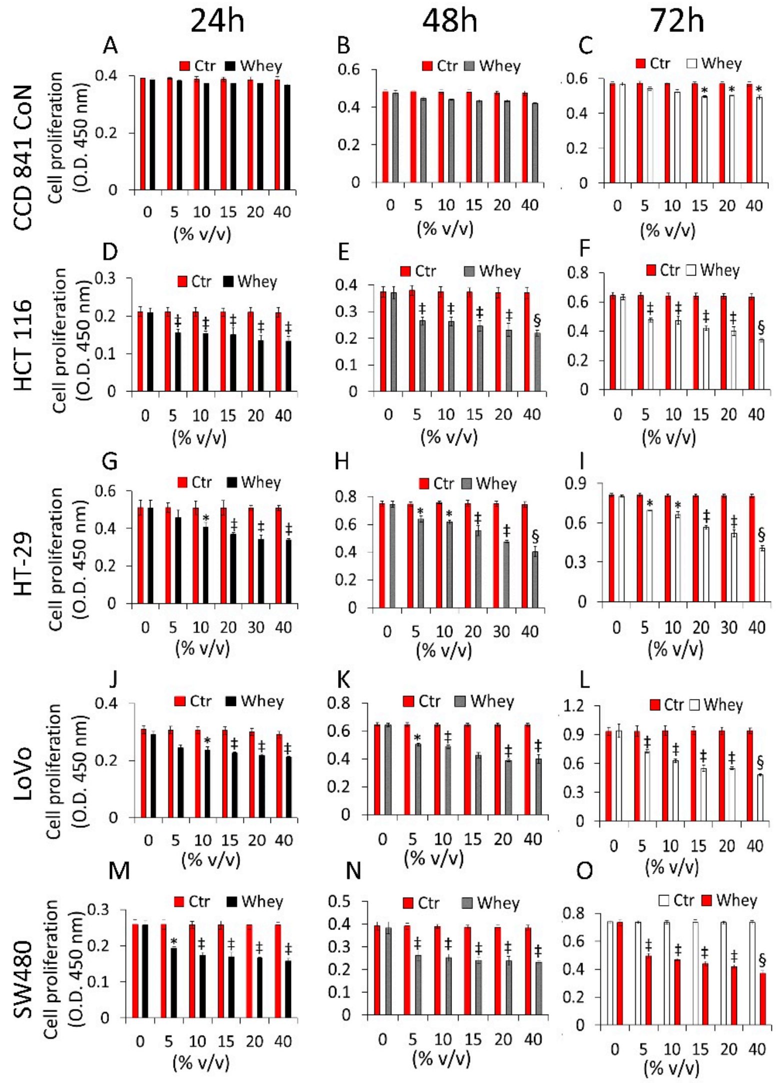

Figure S1. Whey effects on colorectal cell proliferation. Cell proliferation assays after exposure to different volumes (0, 5, 10, 15, 20 and 40 % v/v) of HBSS-10 mM Hepes (Ctr) or whey for different times (24, 48 and 72 h), were performed in (A-C) CCD 841 CoN, (D-F) HCT 116, (G-I) HT-29, (J-L) LoVo and (M-O) SW480 cell lines and expressed as optical density (O.D.) values. Cell proliferation was assessed using Cell Counting Kit-8 assay (Donjindo Molecular Technologies, Tokyo, Japan). Values represent the mean±SD of three independent experiments. *p<0.05 vs. Ctr; ‡p<0.01 vs. Ctr; §p<0.001 vs. Ctr.

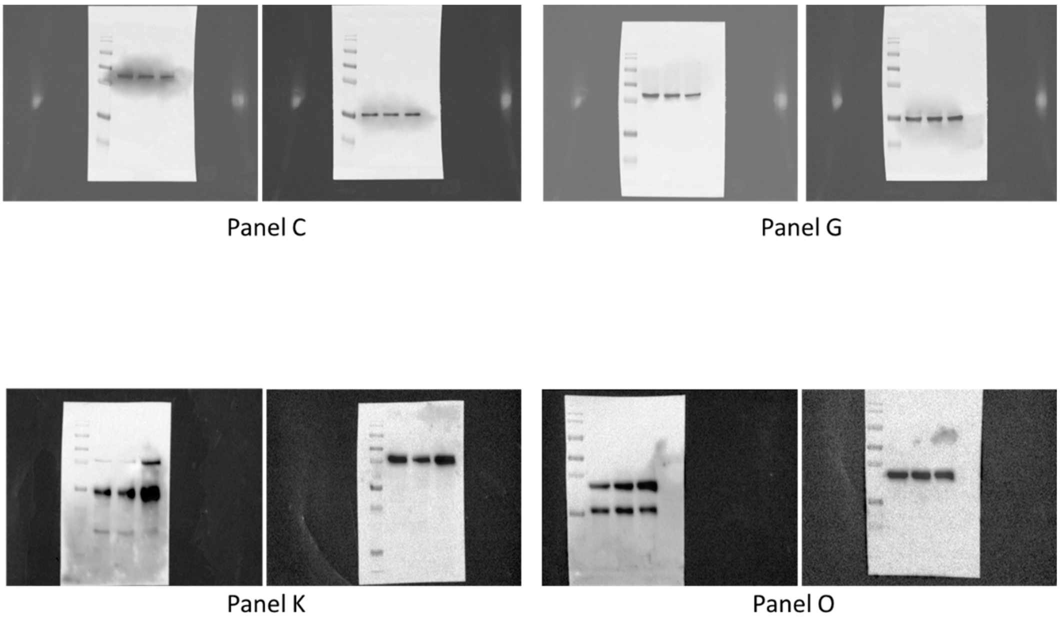

Figure S2. Image representing untouched, uncropped gels related to Figure 2.

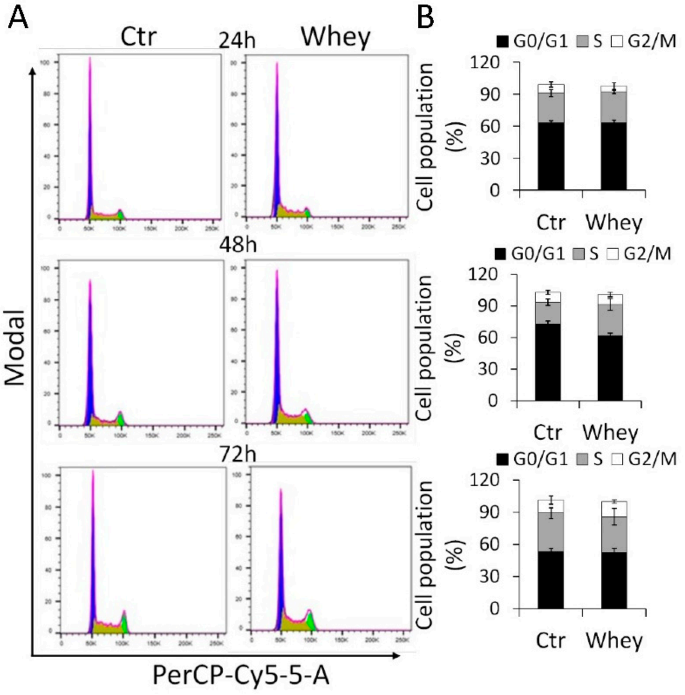

Figure S3. Whey treatment does not modulate the CCD 841 CoN colon cell cycle. (A-B) Representative dot plots and cell cycle analysis of CCD 841 CoN non-tumoral colon cell line. CCD 841 CoN cells were treated with 40 % v/v HBSS-10 mM Hepes (Ctr) or whey 40 % v/v for 24, 48 and 72 h and cell distribution assessed by flow cytometry collecting PI fluorescence as FL3-A (linear scale). For each sample at least 10,000 events were collected and analysis performed by using ModFIT software.

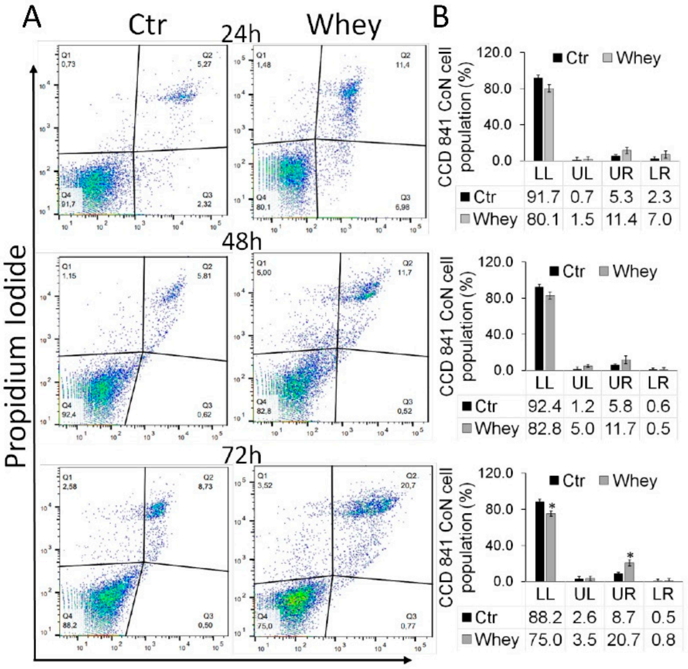

Figure S4. Whey does not display apoptotic effects in CCD 841 CoN cells. (A-B) Representative dot plots and analysis of annexin V-FITC and PI-stained CCD 841 CoN normal colon cells. Cells were treated with 40 % v/v HBSS-10 mM Hepes (Ctr) or whey 40 % v/v for 24, 48 and 72 h and cell viability/death assessed by flow cytometry. Lower left (LL), viable cells; upper left (UL), necrotic cells; lower right (LR), early apoptotic cells; upper right (UR), late apoptotic cells. Data are expressed as mean±SD of n = 3 experiments. At least 10,000 events were acquired. *p<0.05 vs. Ctr.

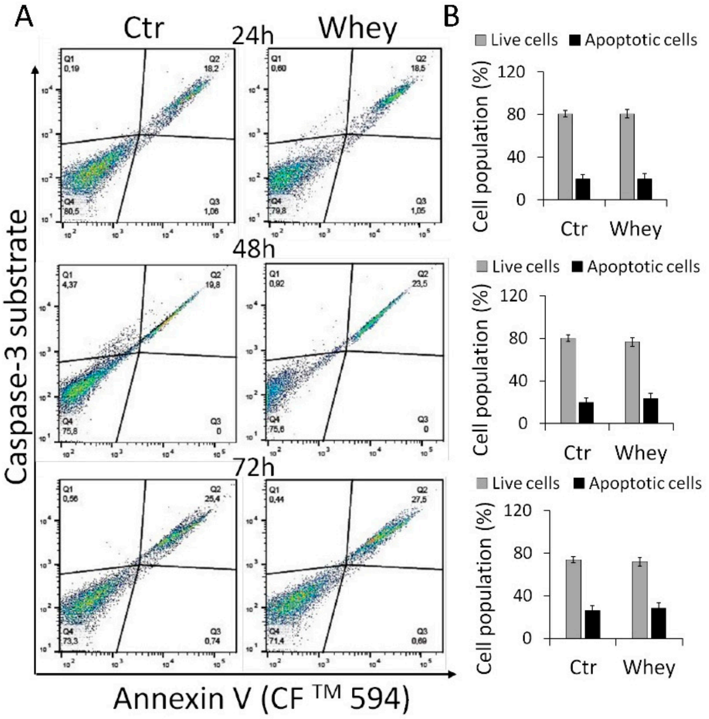

Figure S5. Whey does not mediate caspase-3 activation in non-tumoral colon cells. (A-B) Representative dot plots and analysis of caspase-3 activation in CCD 841 CoN normal colon cells. Cells were treated with 40 % v/v HBSS-10 mM Hepes (Ctr) or whey 40 % v/v for 24, 48 and 72 h and cell population assessed by flow cytometry. Data are expressed as % of live or apoptotic population with mean±SD of n = 3 experiments. At least 10,000 events were acquired.

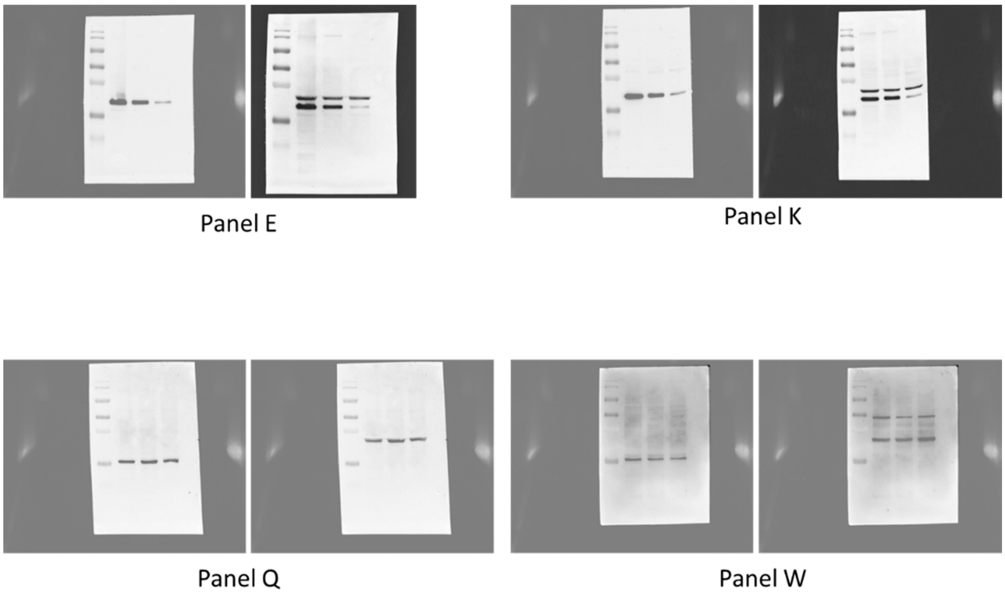

Figure S6. Image reporting untouched, uncropped gels related to Figure 5.

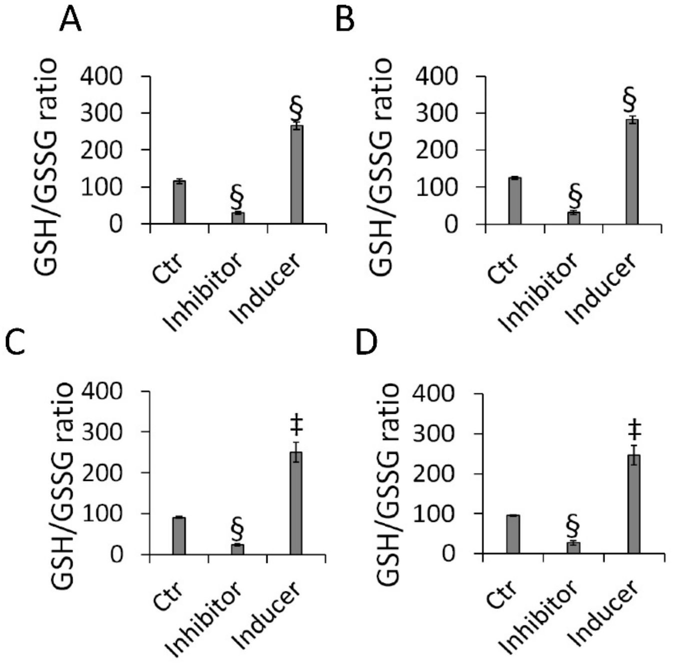

Figure S7. Redox state controls. GSH/GSSG ratios detected on (A) HCT 116, (B) HT-29, (C) SW480 and (D) LoVo cells. Cell lines were treated with 40 % v/v HBSS-10 mM Hepes (Ctr) or 20 µM ethacrynic acid (Glutathione S-transferase inhibitor) or dihydromyristicin (Glutathione S-transferase inducer) for 2 h and redox state assessed by GSSG/GSH quantification kit (G257, Dojindo Molecular Technologies, Tokyo, Japan). ‡p<0.01 vs. Ctr; §p<0.001 vs. Ctr.

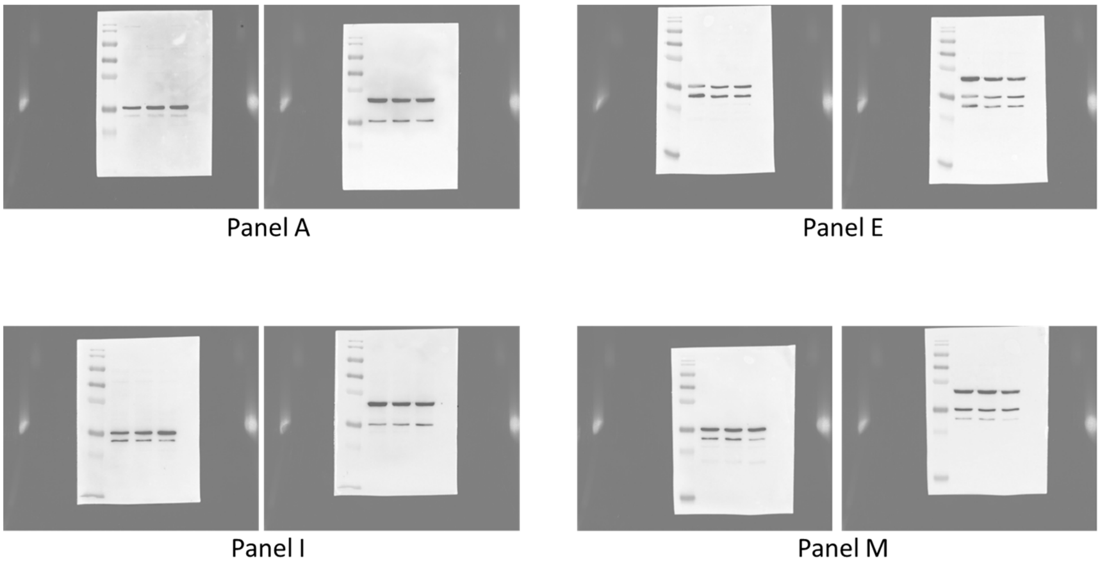

Figure S8. Image reporting untouched, uncropped gels related to Figure 6.

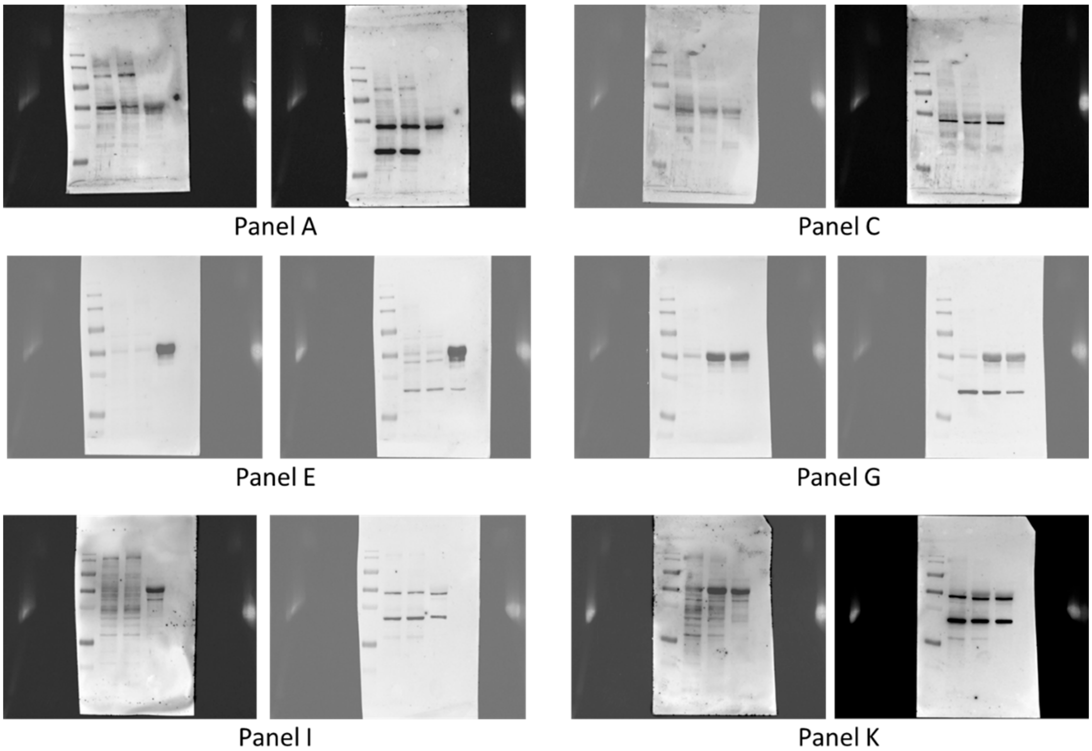

Figure S9. Image reporting untouched, uncropped gels related to Figure 7.

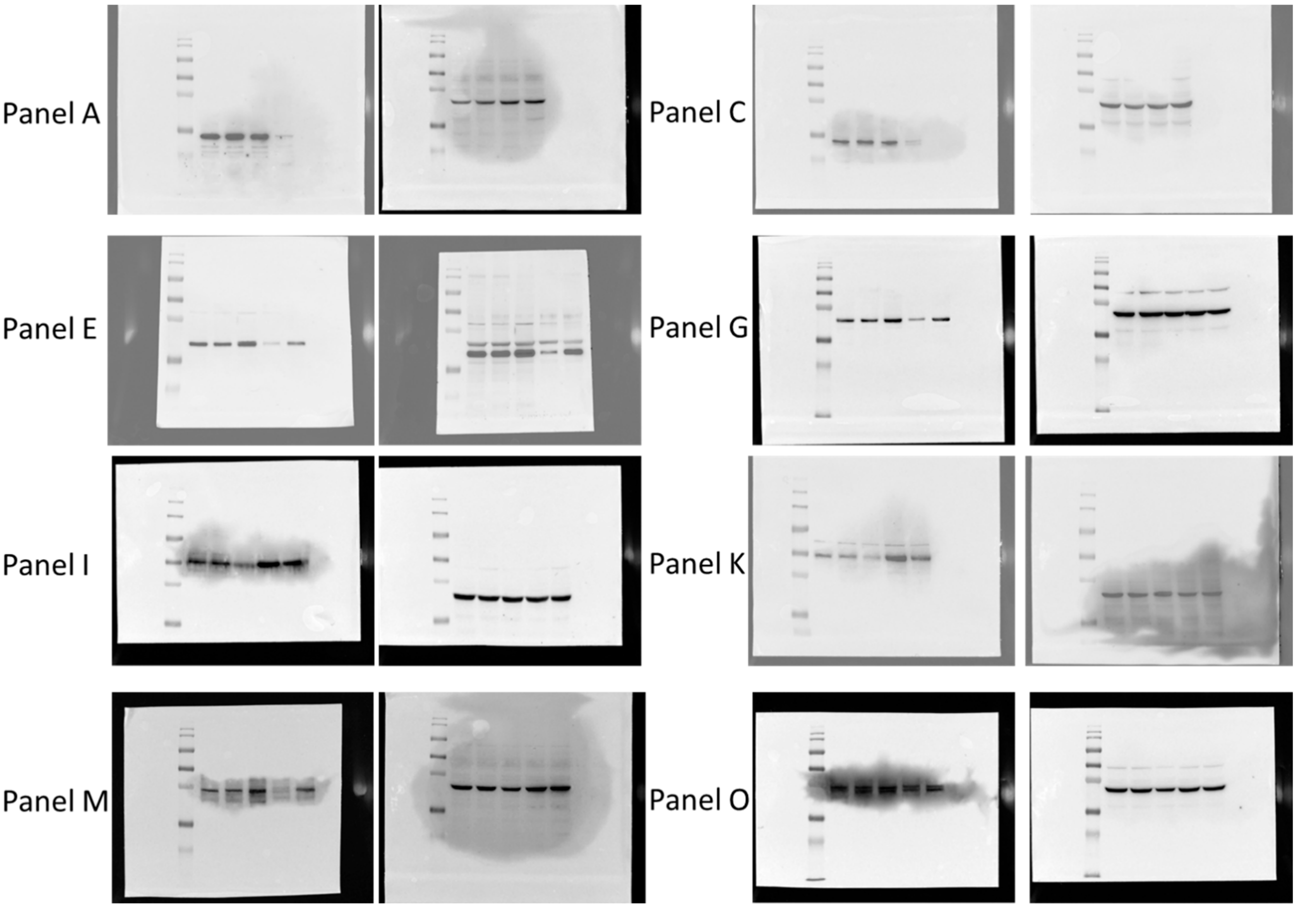

Figure S10. Image reporting untouched, uncropped gels related to Figure 8.

Table S1. Oligo sequences for SIRT3 silencing and negative control (NT-siRNA).

RNA	***Sequence***
SIRNA-SIRT3-OLIGO 1	Sense: 5' – GGUGGAGGAUGGUCCAUAUTT - 3'
	Antisense: 5' – AUAUGGACCAUCCUCCACCTT – 3'
SIRNA-SIRT3-OLIGO 2	Sense: 5' – CCAGCGGCAUCCCAGACUUTT – 3'
	Antisense: 5' – AAGUCUGGGAUGCCGCUGGTT – 3'
SIRNA-SIRT3-OLIGO 3	Sense: 5' – CCCUGACUCAAAGCUCGUUTT – 3'
	Antisense: 5' – AACGAGCUUUGAGUCAGGGTT – 3'
Negative Control	Sense: 5' – UUCUCCGAACGUGUCACGUTT – 3'
	Antisense: 5' – ACGUGACACGUUCGGAGAATT – 3'
